# Robotic-Assisted Single Anastomosis Duodeno-Ileal Bypass with Sleeve Gastrectomy – A Systematic Review

**DOI:** 10.1007/s11695-026-08540-5

**Published:** 2026-02-18

**Authors:** Matthew G. Davey, Noel E. Donlon, William B. Robb

**Affiliations:** 1 Royal College of Surgeons in Ireland, Dublin, Ireland; 2 Blackrock Health Blackrock Clinic, Dublin, Ireland; 3 Beaumont Hospital, Dublin, Ireland

## Abstract

**Background:**

The anticipated surgical and postoperative outcomes following robotic-assisted single anastomosis duodeno-ileal bypass with sleeve gastrectomy (R-SADI-S) are not well described in the surgical literature.

**Aims:**

To perform a systematic review to evaluate clinical and surgical outcomes in patients who have undergone R-SADI-S.

**Methods:**

A systematic review was performed in accordance with the PRISMA guidelines. Basic descriptive statistics were performed using SPSS v26.0.

**Results:**

Overall, 4 studies including data from 160 patients were included. The mean age at the time of surgery was 38.1 years and 55.6% of patients were female (89/160). The mean reported preoperative weight was 122.2 kg and mean body mass index was 45.1 kg/m^2^. The mean time taken for R-SADI-S was 181 min and 7.5% of patients experienced postoperative complications (12/160), however, just 1.4% of patients required either reintervention and readmission (2/144) respectively. At 24-months follow-up, patients who had undergone-R-SADI-S experienced an average weight loss of 60.7 kg, a BMI change of -18.0 kg/m^2^, a total body weight loss of 44.6% and excess weight loss of 113.7%.

**Conclusion:**

R-SADI-S is a seemingly safe procedure which provides excellent weight loss results when performed for patients living with severe obesity. Given R-SADI-S is a novel technique, these results require further ratification once the learning curve is navigated in prospective analyses to ensure the optimisation of patient outcomes.

**Supplementary Information:**

The online version contains supplementary material available at 10.1007/s11695-026-08540-5.

## Introduction

Obesity represents one of the greatest healthcare concerns, with the condition harbouring both direct and indirect negative ramifications on both patient health and healthcare economies [1, 2]. At present, bariatric and metabolic surgery represents the cornerstone in the management of severe obesity [3], with hypoabsorptive approaches, such as the traditional Roux-en-y gastric bypass (RYGB), carrying pragmatism in reducing patient weight, while simultaneously reversing the metabolic sequalae associated with this disease [4]. Moreover, restrictive surgical strategies, such as the sleeve gastrectomy, have proven beneficial in reducing the risk of complications, while also demonstrating efficacy in weight loss [4]. Importantly, contemporary bariatric surgery has evolved and minimally invasive strategies to both restrictive and hypoabsorptive procedures have become popularized with the benefits of smaller incisions, reduced postoperative pain and shorter hospital stays [5]. The non-inferiority of robotic-assisted approaches has also been reported in the literature, with suggested advantages of reducing patient blood loss and infectious complications [6, 7], with improved ergonomics for the operating surgeon [8].

Currently, single anastomosis duodeno-ileal bypass with sleeve gastrectomy (SADI-S) serves as a hybrid hypoabsorptive and restrictive surgical approach and has remained at forefront of the bariatric patient management paradigm for the past decade [9]. SADI-S, which was first described in 2007 by Sanchez-Pernaute et al. [10], was proposed as a means of deploying a pyloric-preserving restrictive approach when compared relative to traditional duodenal switch (DS). SADI-S also serves by having just a single anastomosis with biliopancreatic diversion, while also providing the restrictive benefits observed with sleeve gastrectomy [11]. There is now long-term evidence from the initial series of 164 consecutive patients from Madrid to indicate that SADI-S serves by inducing ‘satisfactory’ weight loss and comorbidity resolution in those living with severe obesity [9].

Notwithstanding these important results, the deployment of the robotic platform in patients indicated to undergo SADI-S for severe obesity provides very promising results, with robust application likely to enhance clinical, surgical and metabolic outcomes. The use of robotic-assisted technology has dramatically improved the anticipated outcomes for patients undergoing several benign and malignant conditions [12–15]. Therefore, it is plausible that the application of robotic surgery may enhance the outcomes for those undergoing SADI-S for severe obesity by simplifying what can be a challenging laparoscopic operation. Accordingly, the aim of the current study was to perform a systematic review to evaluate clinical and surgical outcomes in patients following robotic-assisted single anastomosis duodeno-ileal bypass with sleeve gastrectomy (R-SADI-S).

## Methods

This systematic review was conducted in accordance to the ‘Preferred Reporting Items for Systematic Reviews and Meta-Analyses’ (PRISMA) [16]. This study was prospectively registered with the International Prospective Register of Systematic Reviews (PROSPERO – CRDXXXXXXX). There was no indication for local institutional ethical review / approval for this study as it is a review of the currently available published evidence.

### Search Strategy

Two independent reviewers performed a systematic, electronic search of the PUBMED, Science Direct and Cochrane library databases for relevant studies. This search is outlined in detail in the **Supplementary Material ****1**. Included studies were limited to the English language and were not restricted by year of publication. All duplicates were manually removed, before titles were screened, and studies considered appropriate had their abstracts and/or full text reviewed. Retrieved studies were reviewed to ensure inclusion criteria were met. In cases of discrepancies in opinion among reviewers as to study eligibility, the senior author was asked to arbitrate. The systematic search was performed on the 24th of August 2025.

### Eligibility Criteria

Studies were considered for inclusion if they reported outcomes in relation to patients undergoing R-SADI-S, as outlined within the Population, Intervention, Comparison (PICO) Outcome Framework [17]. Within this, the aspects the authors wished to address were Population (data being presented on adult patients aged 18 years or older who are indicated to undergo R-SADI-S for severe obesity), intervention (any patient indicated to undergo R-SADI-S), comparison: historical comparison between standard laparoscopic SADI-S, and the primary outcomes of interest included intraoperative duration (measured in hours), conversion to open, conversion to right hemicolectomy, reoperation, complications, LOS (measured in days), and estimated costs. Studies had to include more than 15 consecutive patients to warrant inclusion. Studies failing to fulfil this criteria were deemed ineligible and excluded from this study. Furthermore, literature reviews, case reports and studies not published in the English language were excluded.

### Data Extraction

The following data were extracted and collected from retrieved studies meeting inclusion criteria: (1) first author name, (2) year of publication, (3) study design, (4) country of origin, (5) number of patients, (6) patient gender, (7) mean patient age, (8) data pertaining to patient comorbidities, (9) data pertaining to peri-operative data, (10) data pertaining to post-operative data, (11) long-term weight loss data, (12) quality of life data, (13) bowel function postoperatively, (14) reflux symptoms postoperatively and (15) minimal and vitamin deficiencies.

### Statistical Analysis

Descriptive statistics were deployed to detail the outcomes of interest, as appropriate. Standard deviations (SD) were calculated were required. Descriptive statistics were performed using the Statistical Package for Social Sciences (SPSS) version 26 (International Business Machines Corporation, Armonk, New York).

### Risk of bias Assessment

As all included studies were of observation studies of non-randomized design, methodology assessment was undertaken using the Newcastle Ottawa Scale [18].

## Results

### Literature Search

**Fig. 1:**
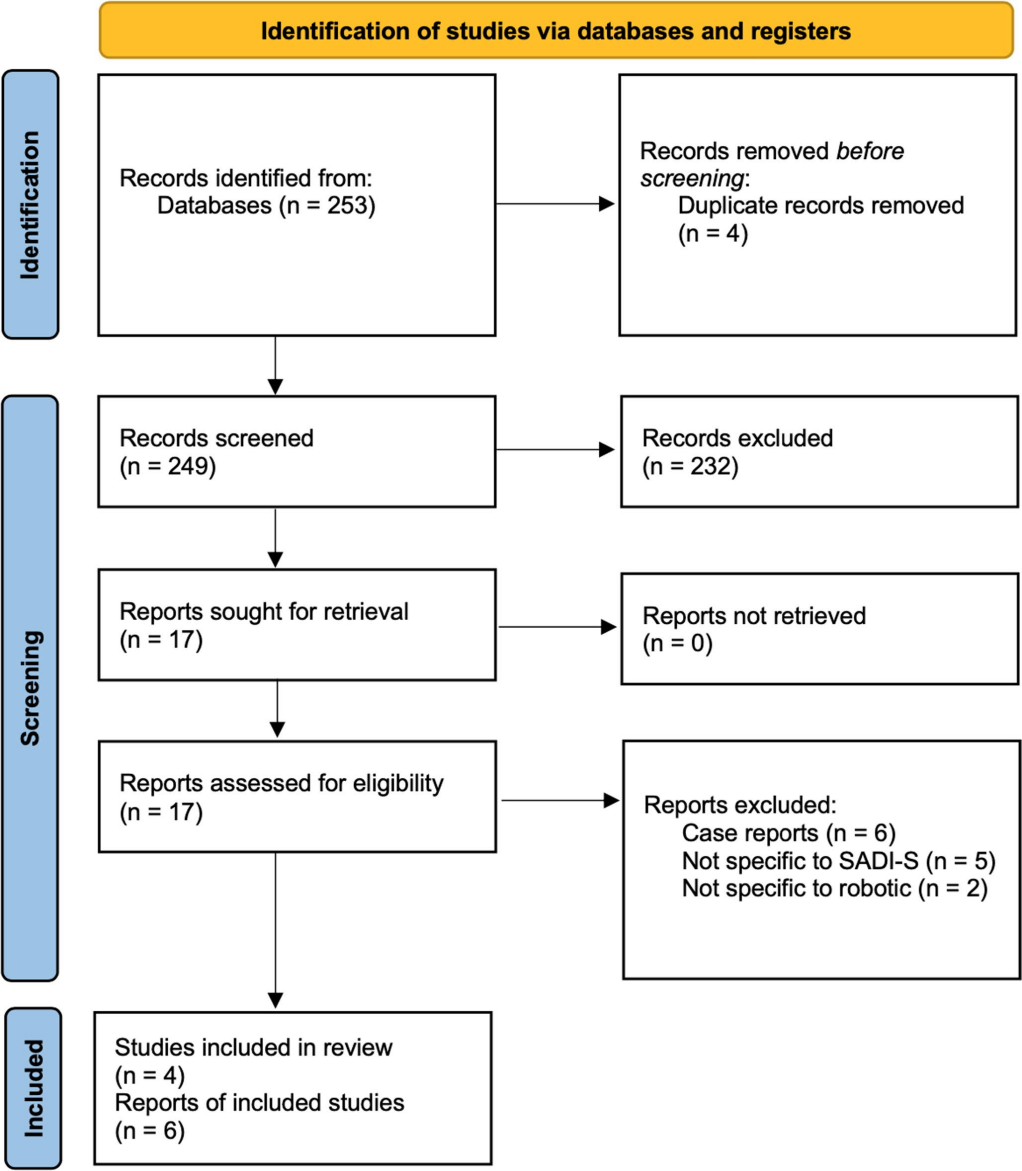
PRISMA flowchart outlining the systematic search process

In total, 253 articles were identified before 4 duplicate articles were excluded. Thereafter, 249 study titles were screened, before 17 abstracts were reviewed for relevance. All 17 of these studies were eligible for full-text review and just 4 of these studies met the eligibility criteria and were included in this systematic review [19–23]. Of note, two reports [22, 24] from the same organisation as included studies were reviewed but excluded (on account of them reporting outcomes from patients who were already captured in the review from another report) [20, 23]. Two case series with less than 15 consecutive patients were identified however did not meet the eligibility criteria [25, 26]. Figure 1 outlines the systematic search process.

### Study Characteristics

All 4 studies were of single centere retrospective observational design, reporting outcomes in relation to patients indicated to undergo R-SADI-S [19–23]. Publication dates of included studies ranged from 2021 to 2024. Study details and risk of bias assessments for the 4 included studies are outlined in Table 1.

**Table 1:** Basic study data from the 4 included studies

Year	Country	Study design	Number	Surgery	NOS
2024	Italy	RCS	26	Primary	4
2023	China	RCS	102	Primary	5
2022	USA	RCS	16	Revisional	3
2021	Spain	RCS	16	-	3

*USA* United States of America, *RCS* retrospective cohort study, *NOS* Newcastle-Ottawa Scale

### Patient Characteristics

Overall, data from 160 patients was included. The mean age at surgery was 38.1 years (SD: 25 years) and 55.6% of patients were female (89/160). The mean reported preoperative weight was 122.2 kg (SD: 6.1 kg) and mean body mass index (BMI) was 45.1 kg/m^2^ (SD: 6.0 kg/m^2^).

Prior to surgery, most patients had American College of Anesthesiologists (ASA) grade II (67.7%, 69/102). Overall, 28.6% and 30.8% had previous diagnoses of obstructive sleep apnea (OSA) (12/42) and type II diabetes mellitus (T2DM) (8/26). In total, 33.3% and 18.8% had previous diagnoses of hypertension (14/42) and hyperlipidemia (3/16).

### Surgery Characteristics

In total, 21.8% of patients had undergone previous abdominal surgery (28/128), including 16 patients who were undergoing R-SADI-S as revisional surgery. The mean operative time taken for R-SADI-S to be performed was 181 min (SD: 40 min). Overall, 7.5% of patients developed postoperative complications (12/160); of these, 6 were classified as major complications (e.g.: 3 leaks (2 gastric and 1 duodeno-ileal), 2 incarcerated internal hernias, and 1 episode of severe respiratory compromise) while 6 were classified as minor complications (e.g.: 2 minor haemorrhage, 2 lower respiratory tract infections, 1 delayed gastric emptying, and 1 seroperitoneum).

Overall, 7.7% of patients necessitated intensive care unit (ICU) admission (2/26). The mean length of hospital stay was 2 days (interquartile range: 2–3 days). In total, just 1.4% of patients required reintervention and readmission (2/144) respectively. One study provided an estimation of hospital costs with R-SADI-S costing approximately 7,997 euro (SD: 873 euro).

### Weight Loss

The estimated mean BMI change at 3 months was − 9.2 kg/m^2^ (SD: 2.5 kg/m^2^) and mean estimated loss in total body weight loss (TBWL) was − 20.6% (SD: 4.4%). Interval changes in weight loss (measured in kg), BMI change, TBWL and excess weight loss (EWL) at 3-, 6-, 12- and 24-months are reported in Table 2. Unfortunately, none of the included studies reported outcomes in relation to quality of life, postoperative bowel function, postoperative reflux symptoms, and postoperative minimal and vitamin deficiencies.

**Table 2:** Estimated interval weight loss data

Change	3-months	6-months	12-months	24-months
Weight (kg)	-26.9	-19.8	-51.3	-60.7
BMI (kg/m^2^)	-9.2	-13.8	-17.7	-18.0
TBWL (%)	-21.9	-32.5	-40.9	-44.6
EBL (%)	-52.8	-76.5	-92.5	-113.7

*Kg* kilograms, *BMI* body mass indices, *TBWL* total body weight loss expressed as a percentage, *EWL* excess body weight loss expressed as a percentage

## Discussion

The SADI-S approach has evolved within the contemporary metabolic surgery paradigm to offer a single anastomotic, hypoabsorptive strategy which also allows patients to benefit from the restrictive effects of gastric volume reduction experienced following sleeve gastrectomy. The purpose of this study was to perform a robust review of current literature surrounding robotic-assisted SADI-S, with the initial ambition of comparing results with conventional laparoscopic-assisted SADI-S (L-SADI-S) to determine the non-inferiority of the robotic approach. Unfortunately, the work of Marincola et al. is the only current study which has been performed to directly provide this comparison, with the three other included studies all reporting data from single arm studies, all of which were of retrospective and observational design. These results, in tandem with the fact that included study data has only been made readily available to the readership from just 2021 onwards, highlights the novelty surrounding the deployment of R-SADI-S in contemporary metabolic and bariatric surgical practice, but also the necessity for further datasets to be accrued and disseminated with the academic community to further establish the true surgical safety and efficacy of this approach.

In this study, R-SADI-S provided excellent weight loss over the two-year period for which follow-up was available: At 24-months, patients who had undergone R-SADI-S experienced an average of 60.7 kg weight loss, a BMI change of -18.0 kg/m^2^, a TBWL of 44.6% and EWL of 113.7%. While these studies have demonstrated results which are obviously excellent in reducing patient adiposity, it is also reasonable to surmise that R-SADI-S is important in the reversal of the metabolic sequalae of obesity (i.e.: reversal of OSA, T2DM and Hyperlipidemia), albeit not directly demonstrated in the results of any of the included studies.

Notwithstanding the important results in relation to R-SADI-S and weight loss, these data may be somewhat scrutinized for being limited by failing decipher the effect of R-SADI-S upon quality-of-life metrics. For patients living with obesity, it is well recognised that obesity is associated with significant mental health issues such as anxiety and depression [27, 28], and metabolic surgery has been previously described to positively impact these conditions [29, 30]. The data integrated from these 160 patients failed to outline the psychological impact of surgery upon these patients. Furthermore, the authors suggest that subsequent studies may also focus upon other important metrics, including impact of surgery upon postoperative bowel function, reflux symptoms and vitamin/mineral deficiency secondary to gut hypoabsorption following biliopancreatic diversion.

Importantly, this review highlighted the surgical safety of R-SADI-S as just 7.5% of patients developed a post-operative complication (12/160), with just two necessitating reintervention (1.4%). While the post-operative complication rate may be slightly higher than anticipated for this procedure, it is important to note that one study included data on revisional R-SADI-S and that the learning curve is unlikely to be fully navigated in these small series. Moreover, and albeit nuanced via indirect comparison, the results for reoperation are consistent with the previous literature following laparoscopic-assisted SADI-S (L-SADI-S), where a reoperation rate of 2.7% was observed in a meta-analysis of 1,704 patients undergoing L-SADI-S performed by Verhoeff et al. [31]. Furthermore, the current data, although limited, indicates at least comparable complication rates associated with R-SADI-S compared to L-SADI-S, where a complication rate of 9.8% was observed [31]. In the current study, 1.9% of patients developed a post-operative leak (3/160) which is comparable with this previous meta-analysis in that those undergoing L-SADI-S experience a 1.8% leak rate) [31]. While post-operative results are consistent with L-SADI-S, a significant difference in intraoperative time certainly distinguishes these procedures; the mean intraoperative time for R-SADI-S was 181 min compared to just 103.6 min for those undergoing L-SADI-S in the review by Verhoeff et al. [31]. This may be reasonably explained by the time utilised to safely ‘dock’ and setup the robotic platform prior to commencing the procedure, as well as a likely learning curve associated with a novel procedure. Importantly, similar 24-month weight loss data was observed in this study when compared to the 28-month weight loss data provided for L-SADI-S in the meta-analysis performed by Verhoeff et al. [31]: Overall, the weight loss associated with R-SADI-S was 60.7 kg (vs. 50.9 kg for L-SADI-S), a BMI change of -18.0 kg/m^2^ (vs. -19.8 kg/m^2^ for L-SADI-S) and a TBWL of 44.6% (vs. 37.3% for L-SADI-S). Thus, it is reasonable to suggest that similar weight loss outcomes can be expected following R- and L-SADI-S procedure for patients living with severe obesity.

These aforementioned advantages are of course important points in the peri- and intra-operative decision making surrounding the surgical safety of R-SADI-S in improving patient outcomes within clinical practice. The data presented in this study coherently indicates this procedure should be considered for those with the necessary skills and may serve in improving postoperative outcomes for prospective patients (i.e.: reduced reoperation rates with a comparable incidence of leaks). Furthermore, the strengths of this study include the integration of ‘real world’ data to indicate R-SADI-S deployment may be safe in the context of severe obesity, rendering it a reasonable option for the metabolic surgeon, who has robot skills in their armamentarium. Unfortunately, this study of course has certain unavoidable limitations. Firstly, given the novelty of R-SADI-S, this study comprises data from just 160 patients who underwent R-SADI-S for severe obesity thus significantly limiting the robustness of results. Secondly, this study consists of data from four observational studies of retrospective design thus rendering them subject to ascertainment, selection, and confounding biases. These biases are unfortunately unavoidable. Moreover, the cut-off for selection of studies with 15 patients or less may be scrutinized as being arbitrary and may incur a selection bias, however, this was established to ensure some level of learning curve had been mitigated by operating surgeons. Thirdly, data specific to metabolic surgery should not be limited to weight loss and perioperative outcomes; unfortunately, there was no data available to perform robust analyses on reflux, bowel function, micronutrient levels, or the impact of surgery of quality-of-life metrics. This is disappointing and the authors would advocate for future studies to consider these important outcome measures in this context. Finally, as outlined, this study failed to identify more than one study making direct comparisons between L- and R-SADI-S meaning direct pairwise analyses could not be performed; therefore, the descriptions of comparisons of R-SADI-S with conventional L-SADI-S are inferred from previous published data and not from novel data yielded from the current study [31].

Within the context of contemporary surgery, future directions will most certainly see the expansion of the robotic platform into the field of metabolic and bariatric surgery. Recent evidence has illustrated the increased deployment of robotic surgery in this field, with the work of Bauerie et al. demonstrating an almost threefold increase in robotic surgery between 2015 and 2020 from the Metabolic and Bariatric Surgery Accreditation and Quality Improvement Program (MBSAQIP) database [32]. While R-SADI-S is seemingly in its infancy (this study has data from just 160 patients) and surgeons mitigate the theoretical learning curve associated with novel procedures, sequential reviews of this topic will likely provide further clarity as to the efficacy and safety of R-SADI-S for future patients.

In conclusion, this systematic review highlights the excellent weight loss results which can be anticipated after performing R-SADI-S for patients living with severe obesity. Furthermore, these data highlight the seemingly surgical safety of this procedure when performed by surgeons capable of performing this procedure. Notwithstanding these important results, R-SADI-S is a novel technique which currently remains in its infancy and requiring further ratification in prospective analyses following navigation of the procedure learning curve to ensure the optimisation of patient outcomes.

## Supplementary Information

Below is the link to the electronic supplementary material.

Supplementary Material 1

## Data Availability

No datasets were generated or analysed during the current study.
