# Supplementary material for: Robotic-Assisted Single Anastomosis Duodeno-Ileal Bypass with Sleeve Gastrectomy – A Systematic Review
Source: Obes Surg. 2026 Feb 18;36(3):1370–6. doi: 10.1007/s11695-026-08540-5 (PMC13038633; doi:10.1007/s11695-026-08540-5)
Supplement: Supplementary file 1 — Supplementary Material 1 [file 11695_2026_8540_MOESM1_ESM.docx]

**Robotic-Assisted Single Anastomosis Duodeno-Ileal Bypass with Sleeve Gastrectomy – A Systematic Review**

**Supplementary Material 1**. Systematic search terms.

(("robot"[All Fields] OR "robot s"[All Fields] OR "robotically"[All Fields] OR "robotics"[MeSH Terms] OR "robotics"[All Fields] OR "robotic"[All Fields] OR "robotization"[All Fields] OR "robotized"[All Fields] OR "robots"[All Fields] OR (("robot"[All Fields] OR "robot s"[All Fields] OR "robotically"[All Fields] OR "robotics"[MeSH Terms] OR "robotics"[All Fields] OR "robotic"[All Fields] OR "robotization"[All Fields] OR "robotized"[All Fields] OR "robots"[All Fields]) AND ("assistances"[All Fields] OR "assistant s"[All Fields] OR "assistants"[All Fields] OR "assisted"[All Fields] OR "assisting"[All Fields] OR "assistive"[All Fields] OR "dental assistants"[MeSH Terms] OR ("dental"[All Fields] AND "assistants"[All Fields]) OR "dental assistants"[All Fields] OR "assistant"[All Fields] OR "helping behavior"[MeSH Terms] OR ("helping"[All Fields] AND "behavior"[All Fields]) OR "helping behavior"[All Fields] OR "assist"[All Fields] OR "assistance"[All Fields] OR "assists"[All Fields]))) AND "SADI-S"[All Fields]) OR (("single person"[MeSH Terms] OR ("single"[All Fields] AND "person"[All Fields]) OR "single person"[All Fields] OR "single"[All Fields] OR "singles"[All Fields]) AND ("anastomosis, surgical"[MeSH Terms] OR ("anastomosis"[All Fields] AND "surgical"[All Fields]) OR "surgical anastomosis"[All Fields] OR "anastomosis"[All Fields]) AND "duodeno-ileal"[All Fields] AND ("bypass"[All Fields] OR "bypassed"[All Fields] OR "bypasses"[All Fields] OR "bypassing"[All Fields]))
